# Dr. Charles Gray Cole

**Published:** 1917-01

**Authors:** 


**Dr. Charles Gray Cole,** Albany 1897, died at his home in Binghamton, Oct. 27, aged 51, of pneumonia.

